# Supplementary material for: Modeling glioblastoma heterogeneity as a dynamic network of cell states
Source: Mol Syst Biol. 2021 Sep 16;17(9):e10105. doi: 10.15252/msb.202010105 (PMC8444284; doi:10.15252/msb.202010105)
Supplement: Supplementary file 5 — Source Data for Figure 3 [file MSB-17-e10105-s001.zip › Figure3A_sourcedata/GSEA_3065/nosigdb_state4.GseaPreranked.1622718430994/LARSSON_STATE_2.html]

Details for gene set LARSSON\_STATE\_2[GSEA]

|  || Dataset | state4 |
| Phenotype | NoPhenotypeAvailable |
| Upregulated in class | na\_neg |
| GeneSet | LARSSON\_STATE\_2 |
| Enrichment Score (ES) | -0.7653892 |
| Normalized Enrichment Score (NES) | -2.7090957 |
| Nominal p-value | 0.0 |
| FDR q-value | 0.0 |
| FWER p-Value | 0.0 |
Table: GSEA Results Summary

  

Fig 1: Enrichment plot: LARSSON\_STATE\_2      
 Profile of the Running ES Score & Positions of GeneSet Members on the Rank Ordered List

  

| PROBE | GENE SYMBOL | GENE\_TITLE | RANK IN GENE LIST | RANK METRIC SCORE | RUNNING ES | CORE ENRICHMENT || 1 | NEAT1 |  |  | 41 | 0.315 | 0.0112 | No |
| 2 | GPM6B |  |  | 346 | 0.166 | -0.0285 | No |
| 3 | MEG3 |  |  | 756 | 0.108 | -0.0886 | No |
| 4 | SLC1A3 |  |  | 1069 | 0.083 | -0.1343 | No |
| 5 | DDR1 |  |  | 1941 | 0.048 | -0.2726 | No |
| 6 | ADGRG1 |  |  | 3961 | -0.007 | -0.5989 | No |
| 7 | MIR99AHG |  |  | 4146 | -0.018 | -0.6276 | No |
| 8 | COL4A1 |  |  | 4362 | -0.033 | -0.6606 | No |
| 9 | MIAT |  |  | 4438 | -0.039 | -0.6705 | No |
| 10 | ADGRV1 |  |  | 4457 | -0.041 | -0.6711 | No |
| 11 | LAMP2 |  |  | 4579 | -0.050 | -0.6879 | No |
| 12 | FTX |  |  | 4694 | -0.059 | -0.7029 | No |
| 13 | SPPL2B |  |  | 4698 | -0.059 | -0.7000 | No |
| 14 | LENG8 |  |  | 4798 | -0.068 | -0.7122 | No |
| 15 | SCRG1 |  |  | 4970 | -0.080 | -0.7353 | No |
| 16 | COL4A2 |  |  | 5156 | -0.098 | -0.7597 | Yes |
| 17 | APP |  |  | 5192 | -0.101 | -0.7597 | Yes |
| 18 | DDX17 |  |  | 5227 | -0.104 | -0.7593 | Yes |
| 19 | FGFBP3 |  |  | 5233 | -0.104 | -0.7542 | Yes |
| 20 | PON2 |  |  | 5246 | -0.105 | -0.7502 | Yes |
| 21 | PTN |  |  | 5261 | -0.107 | -0.7464 | Yes |
| 22 | ZNF708 |  |  | 5314 | -0.110 | -0.7485 | Yes |
| 23 | TTC14 |  |  | 5322 | -0.111 | -0.7433 | Yes |
| 24 | GPC1 |  |  | 5348 | -0.113 | -0.7409 | Yes |
| 25 | TNC |  |  | 5359 | -0.114 | -0.7360 | Yes |
| 26 | MARCH6 |  |  | 5370 | -0.115 | -0.7311 | Yes |
| 27 | NCAM1 |  |  | 5391 | -0.117 | -0.7277 | Yes |
| 28 | ATP6AP2 |  |  | 5453 | -0.125 | -0.7305 | Yes |
| 29 | KCNQ1OT1 |  |  | 5539 | -0.135 | -0.7365 | Yes |
| 30 | GPM6A |  |  | 5563 | -0.137 | -0.7325 | Yes |
| 31 | SOX6 |  |  | 5612 | -0.146 | -0.7320 | Yes |
| 32 | KANSL1 |  |  | 5616 | -0.146 | -0.7241 | Yes |
| 33 | PCSK7 |  |  | 5727 | -0.167 | -0.7325 | Yes |
| 34 | ANKRD11 |  |  | 5733 | -0.168 | -0.7237 | Yes |
| 35 | SGCE |  |  | 5743 | -0.171 | -0.7155 | Yes |
| 36 | CANX |  |  | 5755 | -0.173 | -0.7074 | Yes |
| 37 | WSB1 |  |  | 5773 | -0.178 | -0.7001 | Yes |
| 38 | MALAT1 |  |  | 5783 | -0.179 | -0.6913 | Yes |
| 39 | NOVA1 |  |  | 5789 | -0.181 | -0.6818 | Yes |
| 40 | PTPRZ1 |  |  | 5811 | -0.186 | -0.6747 | Yes |
| 41 | SNTG1 |  |  | 5814 | -0.186 | -0.6644 | Yes |
| 42 | LAPTM4A |  |  | 5819 | -0.187 | -0.6544 | Yes |
| 43 | B2M |  |  | 5823 | -0.188 | -0.6442 | Yes |
| 44 | ZNF431 |  |  | 5829 | -0.190 | -0.6342 | Yes |
| 45 | NKTR |  |  | 5853 | -0.195 | -0.6269 | Yes |
| 46 | TMBIM6 |  |  | 5855 | -0.196 | -0.6159 | Yes |
| 47 | MSMO1 |  |  | 5866 | -0.198 | -0.6063 | Yes |
| 48 | ZNF75A |  |  | 5883 | -0.202 | -0.5974 | Yes |
| 49 | TMEM259 |  |  | 5905 | -0.208 | -0.5890 | Yes |
| 50 | HMGCR |  |  | 5906 | -0.208 | -0.5771 | Yes |
| 51 | HACD3 |  |  | 5916 | -0.211 | -0.5666 | Yes |
| 52 | DDX39B |  |  | 5935 | -0.217 | -0.5572 | Yes |
| 53 | SSR4 |  |  | 5941 | -0.218 | -0.5456 | Yes |
| 54 | LUC7L3 |  |  | 5954 | -0.224 | -0.5348 | Yes |
| 55 | RCN2 |  |  | 5987 | -0.234 | -0.5267 | Yes |
| 56 | LAPTM4B |  |  | 5994 | -0.236 | -0.5143 | Yes |
| 57 | MDM4 |  |  | 6003 | -0.239 | -0.5020 | Yes |
| 58 | CREBZF |  |  | 6011 | -0.242 | -0.4894 | Yes |
| 59 | SF1 |  |  | 6015 | -0.244 | -0.4760 | Yes |
| 60 | PRH1 |  |  | 6021 | -0.246 | -0.4628 | Yes |
| 61 | ARGLU1 |  |  | 6023 | -0.247 | -0.4490 | Yes |
| 62 | CALU |  |  | 6049 | -0.268 | -0.4378 | Yes |
| 63 | PGRMC1 |  |  | 6051 | -0.270 | -0.4226 | Yes |
| 64 | DDOST |  |  | 6054 | -0.271 | -0.4075 | Yes |
| 65 | HSPA5 |  |  | 6061 | -0.274 | -0.3929 | Yes |
| 66 | GRIA2 |  |  | 6065 | -0.277 | -0.3776 | Yes |
| 67 | ACSL3 |  |  | 6078 | -0.284 | -0.3634 | Yes |
| 68 | PLAT |  |  | 6079 | -0.285 | -0.3472 | Yes |
| 69 | APLP2 |  |  | 6087 | -0.288 | -0.3320 | Yes |
| 70 | VMP1 |  |  | 6091 | -0.291 | -0.3159 | Yes |
| 71 | LRPAP1 |  |  | 6096 | -0.294 | -0.2998 | Yes |
| 72 | SQLE |  |  | 6123 | -0.316 | -0.2861 | Yes |
| 73 | PDIA3 |  |  | 6124 | -0.317 | -0.2681 | Yes |
| 74 | LMAN1 |  |  | 6130 | -0.322 | -0.2505 | Yes |
| 75 | CALR |  |  | 6153 | -0.344 | -0.2346 | Yes |
| 76 | PRDX4 |  |  | 6155 | -0.345 | -0.2152 | Yes |
| 77 | FUS |  |  | 6170 | -0.371 | -0.1963 | Yes |
| 78 | CD9 |  |  | 6176 | -0.378 | -0.1757 | Yes |
| 79 | SPCS2 |  |  | 6193 | -0.419 | -0.1545 | Yes |
| 80 | MTRNR2L12 |  |  | 6202 | -0.451 | -0.1302 | Yes |
| 81 | BCAN |  |  | 6211 | -0.481 | -0.1041 | Yes |
| 82 | XRCC2 |  |  | 6212 | -0.482 | -0.0768 | Yes |
| 83 | PDIA6 |  |  | 6213 | -0.482 | -0.0494 | Yes |
| 84 | CD63 |  |  | 6220 | -0.496 | -0.0221 | Yes |
| 85 | HSP90B1 |  |  | 6225 | -0.509 | 0.0061 | Yes |
Table: GSEA details [plain text format]

  

Fig 2: LARSSON\_STATE\_2: Random ES distribution      
 Gene set null distribution of ES for **LARSSON\_STATE\_2**
